# Functional Characterization of a L-2-Haloacid Dehalogenase From *Zobellia galactanivorans* Dsij^T^ Suggests a Role in Haloacetic Acid Catabolism and a Wide Distribution in Marine Environments

**DOI:** 10.3389/fmicb.2021.725997

**Published:** 2021-09-21

**Authors:** Eugénie Grigorian, Agnès Groisillier, François Thomas, Catherine Leblanc, Ludovic Delage

**Affiliations:** CNRS, UMR 8227, Integrative Biology of Marine Models, Station Biologique de Roscoff, Sorbonne Université, Roscoff, France

**Keywords:** L-2-haloacid dehalogenase, *Bacteroidetes*, haloacid catabolism, evolution, knockout mutant

## Abstract

L-2-halocid dehalogenases (L-2-HADs) have been mainly characterized from terrestrial polluted environments. By contrast, knowledge is still scarce about their role in detoxification of predominant halocarbons in marine environments. Here, phylogenetic analyses showed a wide diversity of homologous L-2-HADs, especially among those belonging to marine bacteria. Previously characterized terrestrial L-2-HADs were part of a monophyletic group (named group A) including proteins of terrestrial and marine origin. Another branch (named group B) contained mostly marine L-2-HADs, with two distinct clades of *Bacteroidetes* homologs, closely linked to *Proteobacteria* ones. This study further focused on the characterization of the only L-2-HAD from the flavobacterium *Zobellia galactanivorans* Dsij^T^ (ZgHAD), belonging to one of these Group B clades. The recombinant ZgHAD was shown to dehalogenate bromo- and iodoacetic acids, and gene knockout in *Z. galactanivorans* revealed a direct role of ZgHAD in tolerance against both haloacetic acids. Analyses of metagenomic and metatranscriptomic datasets confirmed that L-2-HADs from group A were well-represented in terrestrial and marine bacteria, whereas ZgHAD homologs (group B L-2-HADs) were mainly present in marine bacteria, and particularly in host-associated species. Our results suggest that ZgHAD homologs could be key enzymes for marine *Bacteroidetes*, by conferring selective advantage for the recycling of toxic halogen compounds produced in particular marine habitats, and especially during interactions with macroalgae.

## Introduction

Halogenated metabolites are widely distributed on Earth. They are defined as organic compounds that covalently bind one or several halogen atom(s) such as fluorine, chlorine, bromine or iodine. Over 5,000 different natural organohalogens have been described, mainly from the marine environment (Gribble, 2015). They can be produced abiotically (Dong et al., 2021), but also derived from many different biogenic sources. For example, some chlorinated metabolites are found in land plants and fungi, while brominated metabolites have been mainly related to marine sources such as red and brown algae, or sponges (Amsler, 2008; Bialonska and Zjawiony, 2009; Cabrita et al., 2010; El-Demerdash et al., 2019). Furthermore, during the last century and with industrial developments, man-made polyhalogenated organic products were synthesized and are now persistent organic environmental pollutants (Agarwal et al., 2017; Ang et al., 2018). To mitigate this environmental issue, nature-based solutions are a promising alternative for bioremediation. During the last four decades, research efforts have identified microorganisms that can catabolize diverse mono or polyhalogenated compounds, and characterized a broad range of dehalogenases (Hardman and Slater, 1981; Janssen et al., 1994; Copley, 1998). These enzymes are highly diverse in terms of structure and biochemistry, but they all share the enzymatic capacity of cleaving carbon-halogen bonds. Depending on their catalytic mechanisms and substrates, they are classified in large superfamilies (Janssen et al., 2005; Ang et al., 2018).

The haloacid dehalogenase (HAD) superfamily includes mostly enzymes that hydrolyze carbon-phosphorus or oxygen-phosphorus bonds, such as phosphoesterases, phosphonatases, P-type ATPases, or nucleotidases. Widely conserved among living organisms, they are involved in a variety of cellular processes ranging from amino acid biosynthesis to detoxification (Burroughs et al., 2006). In plant pathogen bacteria, HADs might also be involved in biofilm formation during biotic interactions (Li and Wang, 2011). The “true” dehalogenases of the HAD superfamily are categorized into two types. Type I contains HADs that have an enantioselective dehalogenating activity on D-2-haloacids (D-2-HADs), and those that have non-stereospecific mechanism accepting both D- or L-2-haloacids as substrates (DL-2-HADs). Enzymes from type II specifically act on L-2-haloacids (L-2-HADs) to produce the corresponding hydroxyalkanoic acids with an inverted chirality (Ang et al., 2018). The L-2-HADs are phylogenetically unrelated to D-2-HADs and DL-2-HADs exhibiting a distinct protein fold and catalytic mechanism (Hill et al., 1999).

Many genes encoding L-2-HADs have previously been identified in the genomes of bacteria from contaminated soils or freshwater environments (Copley, 1997; Kurihara and Esaki, 2008). Biochemical and structure/function characterizations of L-2-HAD enzymes have been conducted during the past 30 years to identify new enzymatic or biotechnological processes for detoxification of halogenated pollutants. The large majority of characterized L-2-HADs originate from terrestrial *Proteobacteria* such as DehIVa from *Burkholderia cepacia* MBA4 (Schmidberger et al., 2007), DhlB from *Xanthobacter autotrophicus* GJ10 (Van der Ploeg et al., 1991), or L-DEX from *Pseudomonas* sp. strain YL (Nardi-Dei et al., 1994). The *dhlB* gene has been engineered into land plants by transformation, allowing effective treatment of soil and groundwater contaminated with halogenated solvents (Mena-Benitez et al., 2008). By contrast little is known about L-2-HAD phylogeny, gene abundance and evolution and only one partial phylogenetic tree has been reported to date (Marchesi and Weightman, 2003).

The biological roles of these HADs in terrestrial and/or freshwater bacteria are mostly related to their adaptations to degrade xenobiotic compounds, derived from industrial activities and released in the environment (Copley, 1998; Hill et al., 1999; Janssen et al., 2005). However, sampling efforts for HAD-containing microorganisms mostly focused on polluted matrices, potentially biasing the overall representation of these enzymes. Therefore, the phylogenetic diversity and biological roles of HADs might be underestimated in non-polluted environments (Janssen et al., 2005).

The marine environment contains a large range of natural halogenated compounds, some of them being highly specific and/or potentially toxic, as parts of chemical defenses of marine invertebrates or algae. For instance, while iodinated compounds are relatively rare on Earth, brown macroalgae are main producers of volatile iodocarbons that play a notable role in atmospheric chemistry (Carpenter et al., 2012). Other natural sources of marine halogenated compounds could include volcanoes and organic matter efflux in estuaries (Jordan et al., 2000; Gribble, 2003; Gordon et al., 2015). Very little is currently known about marine dehalogenases, and even less about the metabolic pathways involved in the catabolism of halogenated compounds by marine microorganisms. A recent overview on marine haloalkane dehalogenases related the discovery of 12 new enzymes leading to increase the amount of knowledge on this kind of dehalogenation (Kunka et al., 2018). To date concerning haloalkanoic dehalogenases, only four marine L-2-HADs have been biochemically characterized: DehRhb from a *Rhodobacteraceae* bacterium associated to marine *Polychaeta* (Novak et al., 2013a); two enzymes from the sponge-associated *Paracoccus* sp. DEH99 and *Pseudomonas stutzeri* DEH130 (Huang et al., 2011; Zhang et al., 2013); and one from the psychrophilic strain *Psychromonas ingrahamii* DehPsi (Novak et al., 2013b). The best-studied marine L-2-HAD so far is DehRhb, whose structure and catalytic properties notably differ from soil enzymes (Novak et al., 2013a).

In this context, this paper reports first on the occurrence of L-2-HAD homologous proteins in microorganisms, i.e., marine bacteria and archaea, through large *in silico* genomic surveys and on their phylogeny in the *Bacteroidetes* phylum. It then described the biochemical and functional characterization of the only haloacid dehalogenase identified in the genome of the marine flavobacterium *Zobellia galactanivorans* Dsij^T^. This bacterium has been developed as a laboratory model for marine *Bacteroidetes*, because of its capacities to degrade algal polysaccharides. Its genome also features several genes related to halogen metabolism (Fournier et al., 2014; Barbeyron et al., 2016). Thanks to a recent protocol development (Zhu et al., 2017), a genetic knockout approach was conducted, demonstrating the potential *in vivo* protective role of this L-2-HAD when *Z. galactanivorans* was exposed to toxic halogenated substrates. In addition, the distribution and expression of phylogenetically distinct L-2-HADs were analyzed using metagenomic and metatranscriptomic datasets, highlighting an underexplored marine biodiversity. Finally, the laboratory functional characterization of *Z. galactanivorans* L-2-HAD and environmental survey results on homologous proteins are discussed in light of the biological and ecological roles of these enzymes in marine *Bacteroidetes* in their natural coastal habitats.

## Results

### Phylogenetic Analysis of Microbial L-2-HADs

**FIGURE 1:**
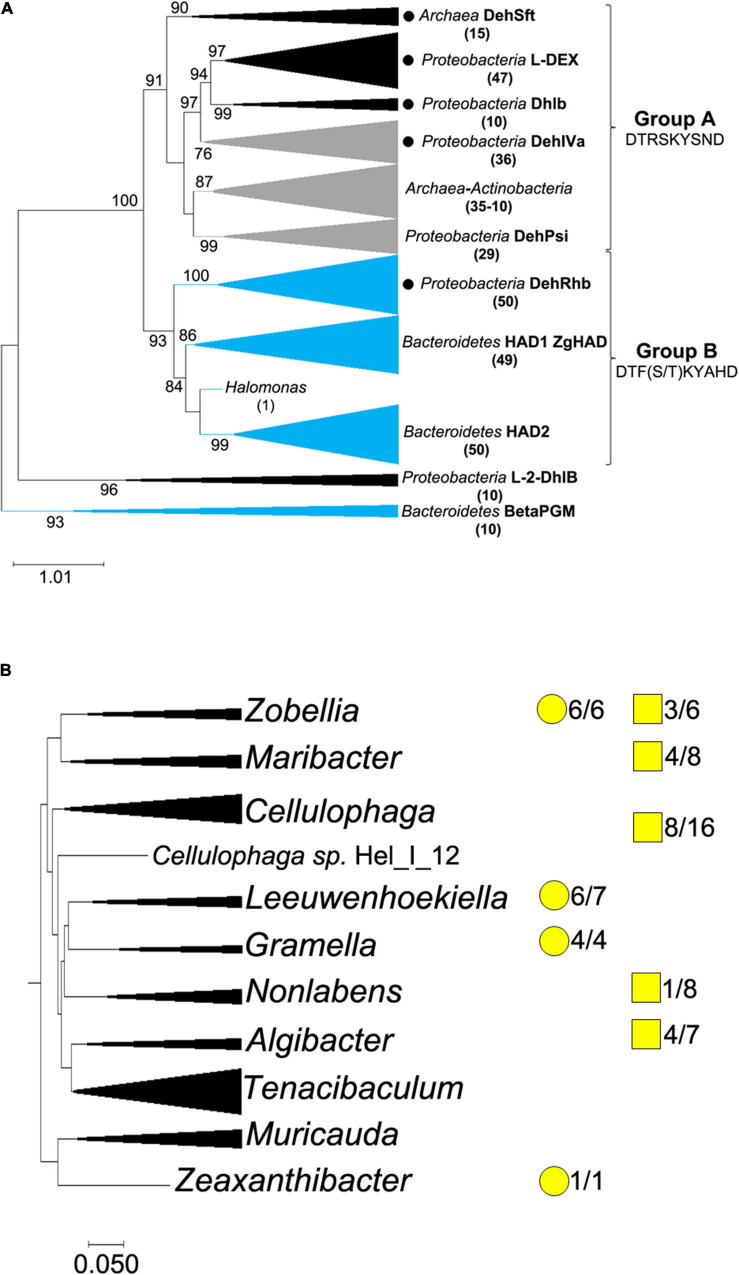
Phylogenetic distribution of L-2-HADs. **(A)** Maximum likelihood phylogenetic tree based on L-2-HAD protein sequences. Group A and group B define the two major phylogenetic groups, for which nine specifically conserved amino acids of the catalytic site are shown. Black circles indicate phylogenetic clades with at least one available 3D structure of L-2-HAD. Black branches represent putative L-2-HAD proteins from terrestrial or freshwater organisms, blue branches represent those from a large majority of marine organisms and gray ones are those for mixed terrestrial/marine groups. The number of sequences in each group is indicated in brackets. DehSft, L-DEX, DhlB, DehIVa, DehPsi, DehRhb, ZgHAD, L-2-DhlB and BetaPGM proteins are indicated next to the clade where they belong. The two distinct clades of *Bacteroidetes* L-2-HADs are named HAD1 and HAD2. The beta-phosphoglucomutase proteins were used as outgroup to root the tree. **(B)** Distribution of the two types of haloacid dehalogenases identified in the family of *Flavobacteriaceae*. The phylogenetic tree of 10 genus clades was constructed using the comparative genomics tool for genome clustering of MaGe website. The ratio numbers correspond to the number of genomes among the total analyzed genomes with homolog genes belonging to HAD1 phylogenetic group (yellow round) and/or to HAD2 phylogenetic group (yellow square). When yellow round and square are not displayed, it means that the corresponding *had* genes were absent.

The phylogenetic analysis of microbial L-2-HAD proteins was based on a subset of 352 protein sequences, including the biochemically and structurally characterized enzymes (Supplementary Table 1) and their closest counterparts, in addition to the closest relatives of ZgHAD, identified by BlastP search on protein databases (Supplementary Table 2). The L-2-HAD proteins mainly clustered into two well-supported groups (Figure 1A), with an additional small clade of proteobacterial sequences related to the L-2-DhlB of *Ancylobacter aquaticus* UV5. The first group (group A) mostly comprises proteobacterial L-2-HAD sequences (122/182 sequences) that are divided into four clades, respectively containing the biochemically characterized L-2-HADs L-DEX, DhlB, DehIVa and DehPsi. Two other clades in the group A contained archaeal proteins: one features terrestrial extremophile L-2-HADs together with the DehSft protein; the second clade contains a mix of *Euryarchaea* and *Actinobacteria* putative L-2-HADs. Most L-2-HAD homologs from the group A have been identified in species of terrestrial or freshwater origins, but also few in marine species (Supplementary Table 2). By contrast, the second phylogenetic group (group B) contains, almost exclusively (138/150 sequences), L-2-HAD protein sequences belonging to microorganisms either from saline or from marine origins (Supplementary Table 2). The group B is constituted of three well-supported clades and an isolated proteobacterial putative L-2-HAD from *Halomonas* (Figure 1A). A clade groups all other proteobacterial L-2-HAD proteins, including DehRhb. Interestingly, the *Bacteroidetes* L-2-HAD homologs are separated into two phylogenetic groups, named HAD1 and HAD2 clades. No *Bacteroidetes* L-2-HAD was yet biochemically characterized before this study. The two phylogenetic types of *Bacteroidetes* L-2-HADs share between 40 and 45% amino acid identity. The phylogenetic group A (Figure 1A) features most of the biochemically characterized L-2-HADs (Supplementary Table 1), and these enzymes share around 30% of identity at the protein sequence level. Furthermore, a motif of nine amino acids (DTRSKYSND) essential for enzyme activity and substrate binding (Adamu et al., 2016) was strictly conserved between all characterized L-2-HADs of the group A (Supplementary Figure 1). In the group B, DehRhb is the closest biochemically characterized protein homolog of ZgHAD, but they only share 31% of amino acid sequence identity (Supplementary Table 1). However, a motif of nine conserved amino acids [DTF(S/T)KYAHD] is present in all group B L-2-HAD sequences and is equivalent to that found for the group A (Supplementary Figure 1). Eight of those amino acids are strictly conserved and only one amino acid is variable in this pattern corresponding to T124 in DehRhb replaced by S131 in ZgHAD (Supplementary Figure 1).

This phylogenetic analysis showed that most *Bacteroidetes* featured a single L-2-HAD belonging to either the HAD1 or HAD2 phylogenetic clades, but some of them exhibited one L-2-HAD sequence from each clade (Supplementary Table 2). When comparing the complete genomes of *Zobellia* species and closely related members of the *Flavobacteriaceae* family (Figure 1B), some inter- and intra-species variations occur, independently of the phylogenetic relationships: for example, *Zobellia amurskyensis* MAR_2009_138 (formerly *Zobellia uliginosa*; Chernysheva et al., 2019) possesses two different L-2-HAD-encoding genes whereas the *Z. galactanivorans* Dsij^T^ genome only encodes for ZgHAD, belonging to the HAD1 phylogenetic clade. The surrounding genes of the two types of L-2-HAD-encoded genes were also very different (Supplementary Figure 2), in agreement with their distinct phylogenetic clades. The HAD1-type homologous genes were present in the genome of all the six *Zobellia* strains as well as in those of *Leeuwenhoekiella*, *Gramella* and *Zeaxanthinibacter* (Figure 1B). The genomes of other genera (*Maribacter*, *Cellulophaga*, *Algibacter* and *Non-labens*) featured one or two HAD2-type homologous genes, but never a HAD1-type gene. Furthermore, this comparative genome study showed that some genera of *Flavobacteriaceae* like *Tenacibaculum* or *Muricauda* had no L-2-HAD-encoding gene.

### Production, Purification and Enzymatic Characterization of the Recombinant ZgHAD

**FIGURE 2:**
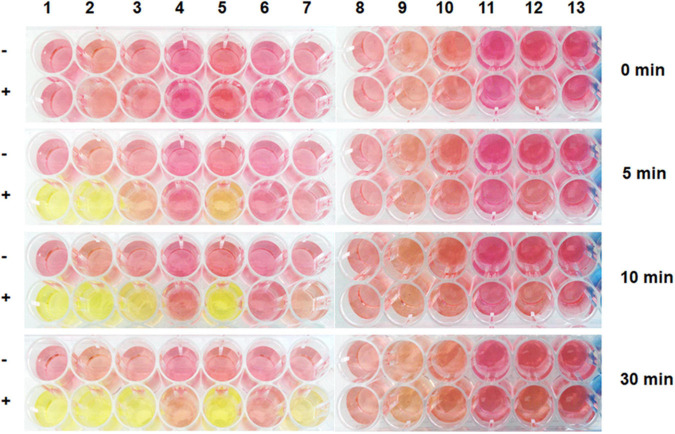
Screening of ZgHAD specificity toward different halogenated substrates based on colorimetric assays. Pictures were taken after 0, 5, 10 and 30 min of enzymatic reaction. Substrates are: (1) iodoacetic acid; (2) bromoacetic acid; (3) chloroacetic acid; (4) D-2-bromopropionic acid; (5) L-2-bromopropionic acid; (6) D-2-chloropropionic acid; (7) L-2-chloropropionic acid; (8) trichloroacetic acid; (9) 3-iodopropionic acid; (10) 3-bromopropionic acid; (11) 2-chlorobutyric acid; (12) iodoacetamide; (13) dibromoacetic acid; (-) no enzyme; (+) with enzyme.

To characterize the biochemical activity of ZgHAD, the recombinant ZgHAD enzyme was produced in *Escherichia coli* and purified to homogeneity (Supplementary Figure 3), yielding 300 mg L^–1^ culture. SDS-PAGE confirmed the purity and correct size of the protein that appeared as a unique band at the expected 26.3 kDa molecular mass (Supplementary Figure 3). Based on size exclusion chromatography, the protein eluted as a homodimer in solution. A standard colorimetric assay was used to first determine the substrate specificity of the recombinant ZgHAD. Thirteen substrates with different carbon chain lengths (C2 to C4) and halogen atom nature, position and number were tested (Figure 2). Within 30 min, the recombinant ZgHAD showed activity only toward L-enantiomer substrates with short carbon chains (C2 and C3). The enzyme was active on substrates that were monoiodinated, monobrominated or monochlorinated on the α-carbon position, namely iodoacetic acid (IAA), bromoacetic acid (BAA), chloroacetic acid (CAA), L-2-bromopropionic acid and L-2-chloropropionic acid (Figure 2). The fastest activity was observed with IAA and BAA, leading to an acidic yellow coloration within 5 min. The chloroacetic acid (CAA) and the L-2-bromopropionic acid were turned partially into their corresponding alcohols at the same time, giving an orange coloration. They were totally transformed between 10 and 30 min (Figure 2). The conversion of the L-2-chloropropionic acid was achieved after more than 30 min. Longer incubations up to 16 h did not show activity on the eight other substrates (data not shown), confirming that the D-2-haloacids, multi-chlorinated or brominated acetic acids, and the halobutyric acids cannot be processed by the recombinant ZgHAD. To further compare the catalytic properties on iodinated and brominated compounds, IAA and BAA were used as substrates to calculate kinetic parameters of ZgHAD. The Vmax and Km value with IAA were 1.12 μM s^–1^ and 0.31 mM, respectively and with BAA they were 1.70 μM s^–1^ and 0.46 mM, respectively.

### Haloacetic Acid Effects on the Growth of Different Bacterial Strains

**FIGURE 3:**
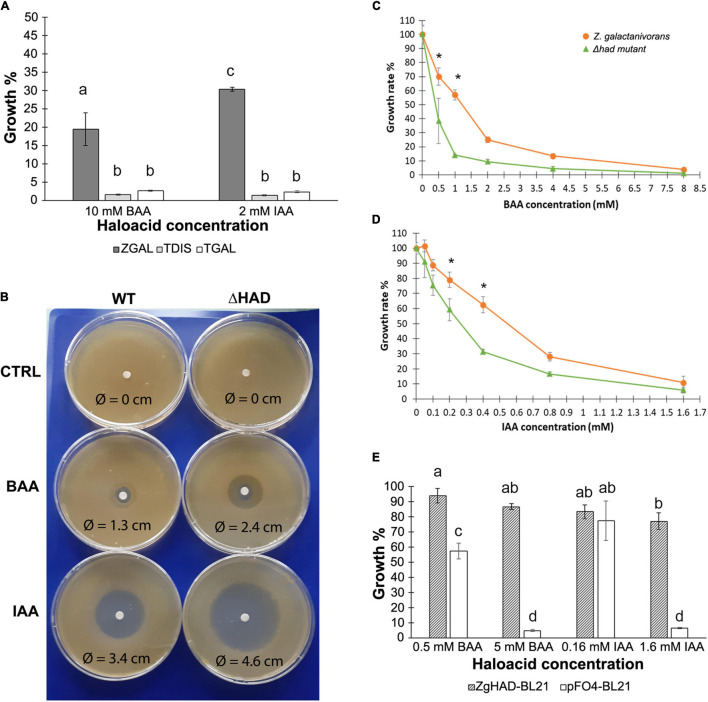
Comparison of bacterial growth of different marine strains, *Z. galactanivorans* HAD knockout mutant and *E. coli* recombinant clones in presence of haloacetic acids **(A)** Growth percentage of *Tenacibaculum* strains (*T. discolor:* TDIS and *T. gallaicum:* TGAL) compared to *Z. galactanivorans* wild type (ZGAL) in liquid cultures supplemented with 10 mM bromoacetic acid (BAA) and 2 mM iodoacetic acid (IAA) (*n* = 3). **(B)** *Z. galactanivorans* WT and Δ*had* growth on solid medium with BAA and IAA solutions deposited on a filter at the center of the plate. The diameter of inhibition around the disk was measured for each plate after 3 days of incubation at 20°C. CTRL: negative control (see also Supplementary Figure 4). **(C,D)** Growth rates of *Z. galactanivorans* wild-type and Δ*had* mutant in liquid cultures with different concentrations of BAA **(C)** and IAA **(D)**. *Z. galactanivorans* (orange line, round dots) and Δ*had* mutant (green line, triangle dots) (*n* = 3). **(E)** Growth percentage in liquid cultures supplemented with either 0.5 or 5 mM BAA and 0.16 or 1.6 mM of IAA compared to the unamended medium (100%) of the ZgHAD-expressing recombinant *E. coli* strain (ZgHAD-BL21) and the *E. coli* control strain (pFO4-BL21) (*n* = 3). Letters and stars indicate statistically significant differences between treatments **(A,E)** and between WT and Δ*had* mutant strains **(C,D)** for the same culture condition (two-way ANOVA, Tukey’s *post hoc* tests, *p* < 0.05).

The growth of *Z. galactanivorans* in the presence of BAA and IAA was compared to that of two *Tenacibaculum* bacterial species (*T. discolor* and *T. gallaicum*), which are phylogenetically closely-related to *Z. galactanivorans* (85.7 and 86.35% 16S rRNA identity respectively) but do not possess any L-2-HAD homolog gene. Bacterial cultures were grown in liquid ZoBell medium with the highest concentrations of BAA (10 mM) or IAA (2 mM) tolerated by *Z. galactanivorans*. After 16 h, cell densities of both *Tenacibaculum* strains were <5% in presence of BAA and IAA compared to unamended ZoBell medium. By contrast, the cell densities of *Z. galactanivorans* WT were significantly higher, and still 20% with BAA and 30% with IAA compared to the unamended ZoBell condition (Figure 3A). This increased tolerance to BAA and IAA in *Z. galactanivorans* compared to the HAD-devoid *Tenacibaculum* strains suggested a potential role of ZgHAD for haloacid metabolism.

To test this hypothesis, we investigated the effect of the *zghad* gene deletion on the *Z. galactanivorans* tolerance to haloacids. On solid medium, BAA or IAA induced clear growth inhibition zones for the wild-type strain (WT), with apparent diameters of 1.3 and 3.4 cm, respectively (Figure 3B). No inhibition zone was observed with the buffer alone. Compared to the WT, the inhibition zones observed for the *zghad*-deleted strain, Δ*had*, were larger, showing an increase of 85% for the BAA (apparent diameter 2.4 cm) and 35% for the IAA (4.6 cm). Consequently, the deletion effect appeared to be more pronounced with BAA than IAA. To confirm these qualitative observations, suggesting a more pronounced *had* deletion effect with BAA than IAA, we compared the growth of the WT and Δ*had* strains in liquid ZoBell medium supplemented with increasing concentrations of BAA or IAA, until reaching the upper toxic limit. For both halogenated compounds, all the culture conditions showed an apparent reduced growth rate for the Δ*had* mutant compared to the WT but only two concentrations are statistically significative (0.5 and 1 mM BAA; 0.2 and 0.4 mM IAA, respectively, in Figures 3C,D). The most significant effect of the *zghad* deletion was obtained with 1 mM BAA where the growth of the WT strain was still 60% and that of the Δ*had* strain was only 10% compared to the control culture conditions without haloacid. Above 2 mM of BAA, the growth rate of the WT strain dropped at 25%, and reached 15% for 4 mM of BAA, whereas that of the Δ*had* strain remained under 10% of the corresponding control growth rate. The growth rates of both WT and Δ*had* strains were very low after incubation with 8 mM of BAA. More than 2 mM of IAA was lethal for both strains. Similarly, upon IAA incubation, the WT strain growth was significantly higher than the Δ*had* mutant between 0.2 and 0.4 mM of IAA (Figure 3D).

In order to further test the role of ZgHAD in haloacid tolerance, the recombinant *E. coli* expression strain (ZgHAD-BL21) was grown in parallel with a control *E. coli* strain transformed with an empty pFO4 plasmid (pFO4-BL21) (Figure 3E). The control pFO4-BL21 strain did not grow with the upper BAA and IAA concentrations (4.9 and 6.4% relative growth compared to that of an unamended LB medium) while the ZgHAD-BL21 strain was able to reach 86 and 77%, respectively. The pFO4-BL21 strain was also significantly inhibited in the presence of 0.5 mM of BAA with 57% relative growth, compared to the ZgHAD-pFO4 strain, which showed no difference compared to the control (94% relative growth).

### Distribution of the Two L-2-HAD Major Phylogenetic Groups in the Environment

**FIGURE 4:**
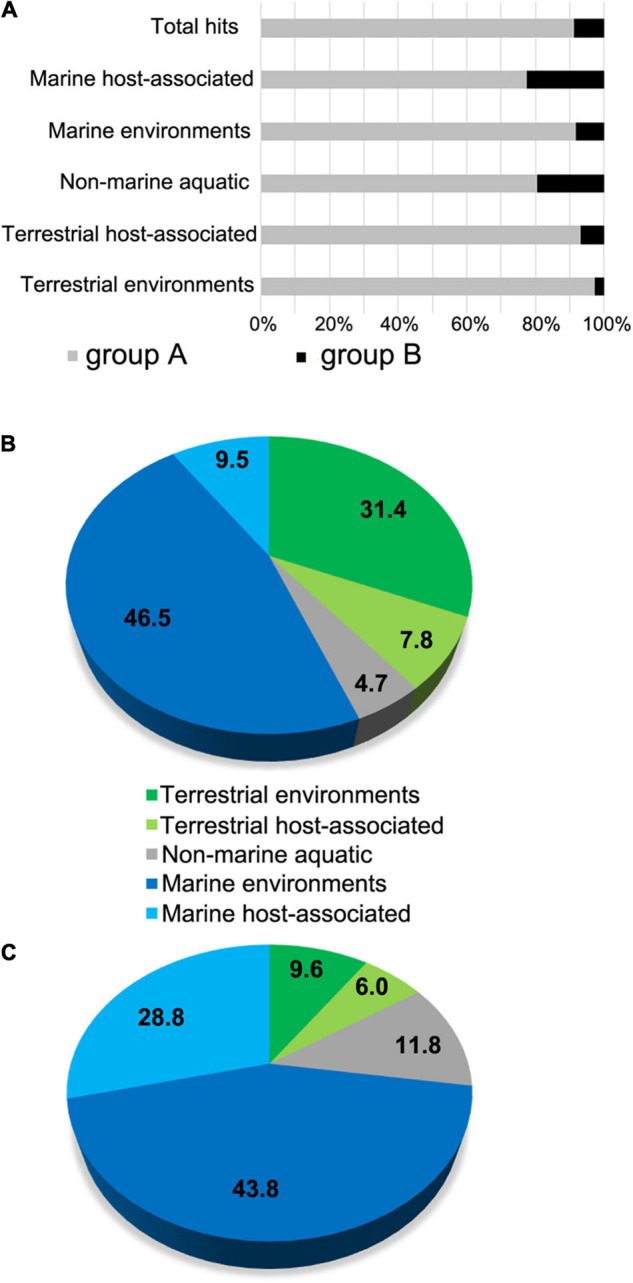
Analysis of environmental distributions of L-2-HAD homologs into IMG/MER metagenome database. **(A)** Global abundance representation of the DehIVa homologs (group A) and ZgHAD homologs (group B) among L-2-HADs hit number in the full set of IMG/MER metagenomes. **(B,C)** Distributions of the group A hits **(B)** and the group B hits **(C)** sequence hits among five IMG/MER ecosystem subsets.

Our phylogenetic analysis suggested that the two different phylogenetic groups A and B, showing different conserved amino-acid signatures, could be differently distributed in the terrestrial and marine environments (Figure 1). To test this hypothesis, we investigated (i) the overall metagenomic abundance distribution of L-2-HAD homologs in contrasted ecosystems using the IMG/MER database (IMG/MER) and (ii) the specific marine distribution of the L-2-HADs through their gene number and gene expression using the Ocean Gene Atlas database (OGA). Among the 25 analyzed ecosystems corresponding to 2,500 metagenomes of IMG/MER (Supplementary Table 3), a total of 4,265 hits were found. The group A L-2-HAD sequences, represented by the DehIVa homologs, were ten times more frequent (3,800 hits) than the group B L-2-HAD sequences (365 hits), represented by the ZgHAD homologs. This preponderance of group A L-2-HADs was verified for all the five major ecosystems of terrestrial, aquatic non-marine and marine habitats with 75–95% of the overall hit identification represented by the group A L-2-HAD sequences (Figure 4A). Nevertheless, the environmental distribution differed in the five datasets (Figures 4B,C). The group A L-2-HAD hits, were almost equally distributed between the non-marine and marine fractions (43.9 and 56%, Figure 4B). By contrast, the majority of the group B hits originated from marine biotopes (72.6% altogether, Figure 4C). As shown in Supplementary Table 3, the group A L-2-HADs are encountered mostly in marine intertidal (764 hits) and coastal (554 hits) zones and in forest soils (560 hits). Moreover, they seemed to be more present in free-living environments (1,768 sequences representing 46.5% and 1,195 sequences representing 31.4% for marine and terrestrial environments, respectively) than in host-associations (9.5 and 7.8% for marine and terrestrial hosts only). By contrast, the group B L-2-HADs were found in bacteria closely linked to marine hosts (28.8% of all the group B hits) compared to terrestrial hosts (6% of hits, Figure 4C). Furthermore, they were particularly found in association with marine macroalgae (76 hits, representing 72.6% of the marine host-associated subset and 20.8% of all the group B hits). Other characteristics of this group B L-2-HADs revealed that these sequences are also principally present in the marine coastal and in the non-marine saline/alkaline environments (51 and 39 hits respectively, 31.9 and 90.7% of their subset, 14 and 10.7% of all the group B hits) (Supplementary Table 3).

**FIGURE 5:**
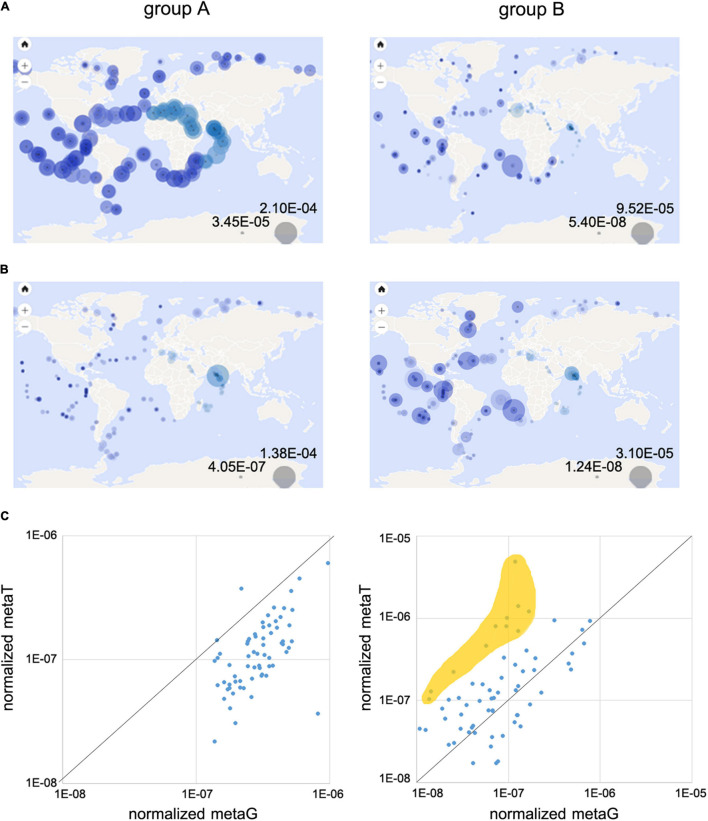
Analysis of oceanic distribution of L-2-HADs into OGA databases. Geographic distribution of relative abundances of group A and group B homologs into metagenomic **(A)** and metatranscriptomic **(B)** OGA databases. Global abundance scales are represented by the two gray circles at the bottom right of the maps. **(C)** Scatterplots of the normalized gene abundance (normalized metaG) vs. normalized transcript abundance (normalized metaT) for each sampling station.

The distribution of the group A and group B L-2-HADs was assessed more precisely in marine environment using Ocean Gene Atlas (OGA, Supplementary Table 4). Interestingly, a similar order of magnitude of the group A sequences was obtained when the marine DehPsi and DehDEH99 proteins were used as queries (166 and 214 hits) while a highest number of hits (about 3–7 fold) was retrieved using the 15 terrestrial sequences. The three selected L-2-HAD protein sequences from the phylogenetic group B (ZgHAD, DehRhb and WP_038235908) allowed to retrieve 204–234 hits (Supplementary Table 4), which all feature the conserved amino acid consensus DETF(S/T)KYAHD. The group A hits were detected in the metagenomes of 66 stations worldwide with an overall gene abundance of at least 10^–5^ (Figure 5A). The group A homologs were more abundant in temperate and tropical zones than in arctic and subarctic zones. By comparison, the group B hits were detected in the metagenomes of 64 stations also well-distributed around the globe but with lower abundances (Figure 5B). We further evaluated the expression of these L-2-HAD homologs by searching hits in the OGA metatranscriptomes. Overall, the representation of group A sequences in metatranscriptomes was low compared to their corresponding detection in metagenomes. The scatterplot of the normalized abundances in metatranscriptomes vs. metagenomes showed that all but one station fell below the 1:1 line, confirming a comparatively low expression of the group A L-2-HAD-encoding genes in most oceanic regions (Figure 5A). Conversely, the metatranscriptomic abundances of group B L-2-HADs were more heterogeneous, and often higher than the corresponding metagenomic abundances for Atlantic and Pacific stations (Figure 5B). The scatterplot demonstrated that the ratio of the group B L-2-HAD-encoding genes abundance in metatranscriptomes vs. metagenomes was ≥1 for 75% of the 64 stations, suggesting a consistent gene expression. In particular, the ratios of 11 stations fell well above the 1:1 line (orange colored zone, Figure 5C), with a metatranscriptome abundance exceeding 5–40 fold that in metagenome. The sample exhibiting the highest expression level for the group B (MetaT/MetaG ratio >40) was located at the station 137 (sample TARA_B100001964, TARA_137_DCM) in the Pacific Ocean at about 500 km off the west coast of Mexico. It corresponds to water collected during late autumn at 40 m depth in the deep chlorophyll maximum layer. The temperature of the water was 20.5°C and pigment detection indicated that chlorophyll a, fucoxanthin, lutein and zeaxanthin were present in the sample. The exploration of individual results for all group B L-2-HAD hits found in this particular sample suggests that mainly two homolog genes (corresponding to OM-RGC.v2.011753063 and OM-RGC.v2.014543634) are highly expressed. Both hits were affiliated to the gammaproteobacterium *Alteromonas australica*.

## Discussion

A bacterial protein named ZgHAD was identified based on its amino acid sequence as the sole L-2-HAD coded in the genome of *Z. galactanivorans* Dsij^T^, a marine bacterium belonging to the *Bacteroidetes* phylum. Although L-2-HAD protein homologs are distributed in numerous bacteria and archaea, only four enzymes have been previously described at the biochemical level from marine bacteria, and little was known about their biological functions, potential ecological role(s) and distribution in marine environment.

The biochemical properties of this newly characterized marine L-2-HAD were compared to other previously characterized L-2-HADs, mostly in *Proteobacteria*. The phylogenetic analysis revealed a poor conservation of amino acid sequences between L-2-HADs from group A and group B. Differences of the two amino acid patterns related to active sites suggested divergent protein folding and biochemical properties. ZgHAD exhibited highest activity toward C2 and C3 carbon-chain substrates with a preference for bromo and iodoacetate, similarly to DehRhb (Novak et al., 2013a), DehIVa (Schmidberger et al., 2007), DehPsi (Novak et al., 2013b) and H-2 (Kawasaki et al., 1981), which used BAA as best substrate. However, ZgHAD did not show any activity toward substrates with a longer carbon-chain or dihalogenated acid substrates, contrasting with other L-2-HADs like DehSft (Rye et al., 2009), DhlB (Ridder et al., 1997), and DehPsi (Novak et al., 2013b) from the phylogenetic group A, suggesting some enzymatic specificities of the marine L-2 HADs belonging to the phylogenetic group B. The Km value of ZgHAD for BAA was 0.46 mM, whereas it varied from 1.1 to 6.72 mM for other L-2-HADs. Notably its closest relative DehRhb showed a higher Km value of 6.72 mM (Novak et al., 2013a) revealing a lower affinity for BAA compared to ZgHAD. The Km of ZgHAD for IAA was lower (0.31 mM) compared to BAA suggesting a higher affinity of ZgHAD for the iodinated substrate. IAA has been previously shown to be a substrate for several L-2-HADs of the phylogenetic group A like DEH99 (Zhang et al., 2014), L-DEX (Liu et al., 1994), DhlS5I (Köhler et al., 1998), and H-2 (Kawasaki et al., 1981), but not for all as shown for DEH130 of *Pseudomonas stutzeri*, isolated from the marine sponge *Hymeniacidon perlevis* (Zhang et al., 2013). However, it is the first time that IAA was reported to be a better substrate than other haloacids for a L-2-HAD. Previous assays with the characterized L-2-HADs did not always include iodinated substrates. As iodide is a better leaving group than bromide or chloride, it is likely that other L-2-HAD enzymes are also active on IAA and therefore on both brominated and iodinated short carbon chains, especially those from marine origin, as these halocarbons are predominant in the marine environment.

**FIGURE 6:**
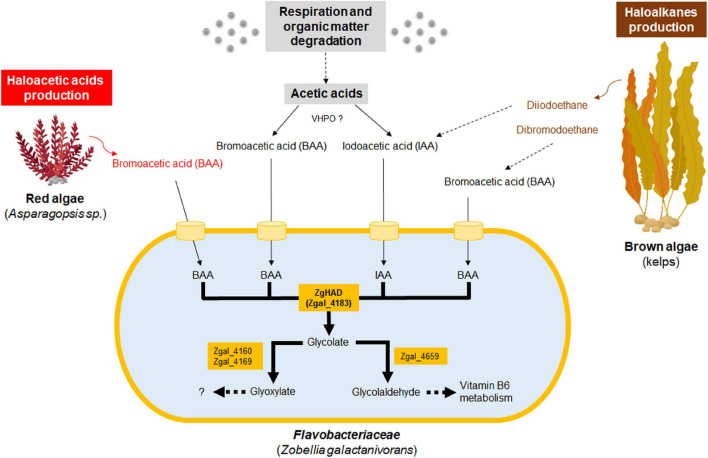
Hypothetical scheme of production and biotransformation of haloacetates by *Z. galactanivorans*, in macroalgae-dominated coastal habitats. ZgHAD and Zgal_4183, L-2-Haloacid dehalogenase of *Z. galactanivorans*; BAA, bromoacetic acid; IAA, iodoacetic acid; Zgal_4160, glyoxylate/hydroxypyruvate reductase; Zgal_4169, glyoxylate reductase; Zgal_4659, glycolaldehyde dehydrogenase; VHPO, vanadium-haloperoxidases. Dotted arrows refer to multistep enzymatic transformations. Oceanic biomass, red algal and brown algal transformation and productions are, respectively, colored in gray, red and brown. Specific toxic compounds produced by red and brown algae are also in red and brown fonts. Orange cylinder models the putative BAA and IAA transporters.

*Zobellia galactanivorans* was able to grow in the presence of higher concentrations of BAA and IAA than its Δ*had* knockout mutant and both fish pathogens *T. discolor* and *T. gallaicum*. The strong growth inhibition induced by BAA and IAA in those strains lacking a L-2-HAD gene suggested it could confer a selective advantage to *Z. galactanivorans* in the presence of toxic short haloacetic acids. This potential benefit was in agreement with the improved tolerance of *E. coli* cells to these haloacids, only when expressing ZgHAD. As ZgHAD enzyme was produced inside *E. coli* cells (in the cytoplasm as also predicted for ZgHAD in *Z. galactanivorans*), this result suggested that bacteria could import BAA and IAA into their cytoplasm for detoxification. Soil bacteria such as *Burkholderia cepacia* MBA4 or *Xanthobacter autotrophicus* GJ10 have been studied for their capacity to grow on and metabolize haloacid compounds. They possess the genetic tools to internalize and degrade a large range of organic halogenated toxic compounds (Kwok et al., 2007; Mena-Benitez et al., 2008; Horisaki et al., 2011). In *B. cepacia* MBA4, the dehalogenase gene *dehIVa* and the downstream permease gene *dehIVp* were shown to form an inducible operon that mediates the transformation and uptake of halogenated acids (Yu et al., 2007). No clear haloacid operon, permease gene or equivalent transport system were identified within *Z. galactanivorans* genome. Nevertheless, the detoxification of haloacetic acids was efficient in *Z. galactanivorans* suggesting that an unidentified transport system might exist. L-2-HADs potentially convert haloacetic acids to glycolate through dehalogenation. *Z. galactanivorans* genome encodes predicted enzymes for downstream glycolate catabolism (Zgal_4160/Zgal_4169 and Zgal_4659) as shown in its KEGG metabolic network and schematized in Figure 6. This putative pathway might link BAA/IAA catabolism either to the glyoxylate cycle or to the vitamin B6 metabolism *via* glycolaldehyde (Figure 6). The Δ*had* mutant of *Z. galactanivorans* resisted better to haloacetic acids than the *Tenacibaculum* strains lacking L-2-HAD. Other unidentified genes could therefore be involved in the resistance against haloacetic acids in *Z. galactanivorans*, similarly to what has been demonstrated for the BAA resistance in *E. coli* (Desai and Miller, 2010). Altogether, these functional studies and gene mining in *Z. galactanivorans* suggested a complete degradation pathway of short carbon chain haloacids, downstream of ZgHAD activity (Figure 6). Future transcriptome analysis of WT and Δ*had Z. galactanivorans* in the presence of BAA or IAA could help elucidate the metabolic processes involved in intracellular haloacid catabolism.

All the available *Zobellia* isolates have one or two copies of L-2-HAD-encoding genes in their genome, a trait that might be a selective advantage linked to the presence of short haloacetic acids in their natural environment. Indeed, coastal ecosystems could be the production source of toxic haloacids through various natural processes (Figure 6). First, low-molecular-weight organic acids such as acetic acids are ubiquitous in seawater, produced either *via* respiration, microbial degradation of lipids or photochemical breakdown of dissolved organic matter (Liang et al., 2020). They might be converted to the corresponding halogenated acetic acids abiotically or biotically, as reported previously in terrestrial environments. For example, the decomposition of forest soil organic matter leads to the production of CAA (Matucha et al., 2003; Laturnus et al., 2005), potentially due to the action of fungal and bacterial chloroperoxidases (Wever and Barnett, 2017). In line with this, we hypothesize that the combination of vanadium-haloperoxidases and L-2-HADs could also contribute to the recycling of acetic acids dissolved in seawater through the production of BAA and IAA intermediates (Figure 6). Second, brominated or iodinated compounds are produced by some marine bacteria, and also by a range of marine macro-organisms living in close association with them (e.g., corals, sponges, macroalgae) (Gribble, 2010). In particular, marine macroalgae are known to release organic brominated and iodinated toxic compounds in the seawater (Belkin, 1992; Paul et al., 2006) and also haloacetic acids such as reported for the red alga *Asparagopsis taxiformis* (Woolard et al., 1979; Fenical, 1982). Moreover, haloalkanes like diiodoethane produced by kelps during biotic interactions could be sequentially transformed into haloacetic acids similarly to the dichloroethane degradation found in *Xanthobacter autotrophicus* GJ10 (Van der Ploeg et al., 1991). Since all known *Zobellia* species live with or close to macroalgae, including red algae such as *Delesseria sanguinea* (Barbeyron et al., 2001) and brown algae such as *Fucales* and *Laminariales* (Nedashkovskaya et al., 2004; Martin et al., 2015), it is likely that they regularly experience the release of haloacetic acids or haloalkanes (Figure 6). Another source of haloacetic acids in coastal seawater might also result from anthropogenic activities (e.g., agriculture, water-treatment stations). Chlorine residuals and humic acids of effluents can react with bromide-rich coastal seawaters (0.8 mM) to form BAA (Gordon et al., 2015). Similar reactions might occur for IAA formation, even if seawater contains only 0.3 μM of iodide (La Barre et al., 2010). The catabolic properties of ZgHAD toward both BAA and IAA might reflect the genetic adaptation of *Zobellia* strains to these halogen-rich marine ecological niches, and especially those formed by the coastal macroalgal beds (Figure 6).

As said above, the phylogenetic tree of L-2-HADs revealed two major distinct phylogenetic groups that might correlate to specific catalytic properties. They also correspond to distinct geographical and environmental distributions. The phylogenetic group A contained a majority of terrestrial but also marine sequences and the phylogenetic group B was essentially composed of marine ones. Furthermore, the group B showed a separation of two distinct *Bacteroidetes* L-2-HAD clades, namely HAD1 (including ZgHAD) and HAD2. Comparative genomics in the *Flavobacteriaceae* family showed that these two L-2-HAD types did not have the same genomic context, suggesting independent acquisition and evolution in the different strains, and a yet unexplored biochemical diversity among *Bacteroidetes* L-2-HAD.

This phylogenetic overview was enlarged with environmental analyses through oceanic (OGA) and multi-ecosystemic (IMG/MER) data. Group A L-2-HADs are more widespread than group B sequences, irrespective of the biotopes, being almost equally distributed between terrestrial and marine habitats. Moreover, the analyses of ocean genomic data revealed that these L-2-HADs were more frequent in the marine environment than initially thought, often exceeding group B sequence abundance. The group B L-2-HADs appeared to be less frequent but they are selectively present in marine environments, in particular in close association with macroalgae. Overall, the presence of toxic haloacids in marine environments likely drove the selection and evolution of L-2-HAD genes. Indeed, the strong prevalence of L-2-HADs in metagenomes from macroalgae or hypersaline waters suggests that these enzymes could be involved in the elimination of haloacetic acids in these particular environments. The high abundance of L-2-HAD genes in oceanic stations could reflect strong biomass production spots like microalgal blooms. Metatranscriptomic analyses of OGA data revealed that group A L-2-HADs have a homogeneously low expression level around the globe while group B expression seems to be induced only in specific zones in response to yet-unknown stimuli. For instance, the highest expression of group B L-2-HADs affiliated to the *Alteromonas* at station TARA_137. Chlorophyll a, fucoxanthin, lutein and zeaxanthin detection at the same point might suggest associations between these *Gammaproteobacteria* and microalgae.

## Conclusion

Biochemical and functional characterizations of the sole L-2-HAD encoded in the genome of *Z. galactanivorans* (ZgHAD) have shown its key role in the catabolism of IAA and BAA in this marine *Bacteroidetes*. ZgHAD could confer a selective advantage to live in haloacetic acid-producing niches, such as those formed by coastal macroalgae. An extensive phylogenetic analysis has revealed the occurrence of two sister groups of L-2-HADs, namely group A and group B. When the well-represented group A contains both terrestrial and marine sequences, the group B was mainly composed of marine L-2-HADs and contained two distinct *Bacteroidetes* clades. The search for group A and group B L-2-HADs homologs in environmental databases further confirmed worldwide, but distinct ecological distribution and expression levels of these two L-2-HAD groups, according to geographical origins, suggesting different ecological functions. For instance, the presence of homologs of ZgHAD could be the functional signature of bacterial haloacetic acid catabolism, especially during micro- and macroalgal interactions. Our study further highlighted the abundance and diversity of marine L-2-HADs, especially of those belonging to the phylogenetic group A, but also some other *Bacteroidetes* L-2-HADs, which are still to be functionally characterized. All these bacterial L-2-HADs are expected to be important actors of halogenated compounds biotransformation and halogen biogeochemical cycle in open marine environments.

## Materials and Methods

### Chemicals

Iodoacetic acid (I4386), bromoacetic acid (17000), chloroacetic acid (C19627), D-2-bromopropionic acid (18165), L-2-bromopropionic acid (38551), D-2-chloropropionic acid (306800), L-2-chloropropionic acid (306797), trichloroacetic acid (91233), 3-iodopropionic acid (I10457), 3-bromopropionic acid (101281), 2-chlorobutyric acid (24008), iodoacetamide (I1149), and dibromoacetic acid (242357) were obtained from Merck.

### Bacterial Strains and Plasmids

Bacterial strains and plasmids used in this study are listed in Supplementary Table 5.

### Phylogenetic Analysis

The selected set of L-2-HAD proteins was derived from NCBI BlastP queries against the RefSeq database. Sequences were loaded into the NGPhylogeny.fr “A la carte” pipeline and analyzed as follows. A total of 352 archaeal and bacterial protein sequences were aligned using MAFFT under default parameters then cleaned with trimAl resulting in 165 informative positions over the 400 aligned positions (see Supplementary Table 2 for complete information). A maximum likelihood phylogenetic analysis was carried out using default parameters of the PhyML-SMS tool allowing the best substitution model selection. Bootstrap analysis with 100 replicates (>70%) was used to provide estimates for the phylogenetic tree topology and it resulted in a newick file formatted with the program MEGA v10.1.1 to obtain the corresponding simplified dendrogram tree figure.

The phylogenetic species tree of 10 genus clades was constructed using the comparative genomics tool for genome clustering of MaGe website. See Supplementary Table 5 for complete list of bacterial genomes.

### Genomic Context Decryption

The *had* gene context in *Flavobacteriaceae* was analyzed by synteny reconstruction on the Genoscope Microscope (MaGE) platform. All the bacterial genomes containing a *had* gene, listed in Supplementary Table 5, were then aligned to allow the identification of the conserved neighboring genes (Supplementary Figure 2).

### Environmental IMG/MER Data Analyses

L-2-HAD homologs were searched by BlastP in the environmental metagenomic IMG/MER database at the JGI website^1^, using DehIVa and ZgHAD sequences as the representative sequences of the phylogenetic group A and group B, respectively. A stringent e-value cutoff of 10^–50^ was selected to identify reliable hits of group A and group B with at least 38% amino acid identity in five datasets (Terrestrial environments, Terrestrial host-associated, Non-marine aquatic, Marine environments, Marine host-associated). Each dataset was constituted of five subsets comprising 100 random selected metagenomes (Supplementary Table 3). The BlastP results of group A and group B sequences were expressed as hit number abundances in each dataset and subset, and as overall percentages.

### Oceanic Distribution and Expression in Ocean Gene Atlas Databases

BlastP searches were carried out with 20 representative L-2-HAD sequences (Supplementary Table 4) against the Ocean Gene Atlas (OGA) database^2^, comprising metagenomic OM-RGCv2 + G and metatranscriptomic OM-RGCv2 + T data collections from the Tara Oceans stations (Villar et al., 2018). A stringent e-value cutoff of 10^–30^ was selected in order to cover a majority of each phylogenetic clade (Supplementary Table 4). In a second step, we focused on the DehIVa and ZgHAD BlastP hits, respectively, as representative sequences of the phylogenetic group A and group B, to compare their geographic repartition, relative gene abundance (metaG) and relative gene expression (metaT) levels. All the data for BlastP searches can be downloaded and extracted at the website. Relative abundance values were calculated based on the sum of all the hits found at a precise site and showed on geographical maps. Relative abundance was further normalized by the number of hits in order to evaluate the average gene abundance and gene expression for every OGA stations. Normalized relative abundances of HAD homologs in metagenomes and metatranscriptomes were calculated for each station and depicted as log10 correlation plots of 66 and 64 spots, respectively, for DehIVa (group A hits) and ZgHAD (group B hits) homologs. Finally, environmental parameters were compared using the bubble plot tools available on the OGA website platform.

### Gene Cloning

The ZgHAD-encoding gene sequence (*zgal_4183*) was cloned from the genomic DNA of *Zobellia galactanivorans* Dsij^T^ (Barbeyron et al., 2001), using primers Zgal_4183fw and Zgal_4183rv (Supplementary Table 6). The PCR product was ligated into the pFO4 vector, using *Bam*HI and *Eco*RI restriction sites and the T4 DNA ligase protocol (New England Biolabs). The recombinant vector was transformed firstly into *E. coli* DH5α for sequence verification and secondly into *E. coli* BL21 (DE3) expression strain.

### Overexpression and Purification

The recombinant *E. coli* strain expressing ZgHAD was grown in LB medium containing 100 μg ml^–1^ ampicillin at 37°C to an optical density of ∼1.0, then the temperature was lowered to 20°C for 1 h followed by a 20 h induction of protein production by the addition of 0.5 mM IPTG. After centrifugation (3,000 *g*, 30 min, 4°C), the bacterial pellet was stored at −20°C, then resuspended in 50 mM Tris (pH 7.5), 500 mM NaCl, bovine DNase I (500 U/μl), 0.1 mg/ml lysozyme, Complete Protease^*TM*^ Inhibitor Cocktail (Merck) and 6 mM MgCl_2_ and lysed using a French press and the lysate was centrifuged at 23,000 *g* during 30 min and 4°C. The recombinant protein was purified by a two-step chromatography carried on an ÄKTA Avant purification system (GE Healthcare Life Sciences). The first step was performed on an immobilized nickel affinity HisTrap^*TM*^ column (GE Healthcare Life Sciences) using an equilibration buffer composed of 50 mM Tris (pH 7.5), 500 mM NaCl and 50 mM imidazole and an elution buffer containing 500 mM imidazole instead of 50 mM. Proteins were eluted with a constant gradient of imidazole concentration. The pooled fractions of the elution peak were concentrated on Centriprep devices and the protein sample was injected on a gel filtration HiLoad^*TM*^ 16/600 Superdex^*TM*^ 200 pg column (GE Healthcare Life Sciences). The purified protein was stored in a buffer containing 20 mM Tris (pH 8.0) and 150 mM NaCl. The purity of the recombinant protein was analyzed by SDS-PAGE (Bio-Rad Mini-PROTEAN^®^ precast gels and systems).

### Substrate Specificity and Kinetic Enzymatic Parameters Determination

The ZgHAD enzyme specificity was determined by adapting the colorimetric assay from Holloway et al., 1998. The detection was based on a pH decrease when the halogenated substrate was reduced to a hydroxyalkanoic acid. Upon dehalogenation, the release of halide ions X^–^ decreased the pH inducing a color transition of the phenol red pH indicator (from pink to orange to yellow) that was monitored visually. The assay solution contained final concentrations of 0.3 mg ml^–1^ recombinant ZgHAD enzyme, 1 mM HEPES, 1 mM EDTA, 20 mM sodium sulfate and 56 μM of phenol red (pH 8.2). The assay was adapted for 48-well plates to test 13 different substrates at 10 mM (iodoacetic acid, bromoacetic acid, chloroacetic acid, D-2-bromopropionic acid, L-2-bromopropionic acid, D-2-chloropropionic acid, L-2-chloropropionic acid, trichloroacetic acid, 3-iodopropionic acid, 3-bromopropionic acid, 2-chlorobutyric acid, iodoacetamide, dibromoacetic acid) in 1 ml final volume. The plates were incubated at 20°C during 30 min then photographed and left 16 h at 20°C to check for long-term variations.

The colorimetric assay was further used for measuring enzymatic kinetic parameters toward IAA or BAA as substrates. All reactions were performed in triplicates at 20°C. Each assay was carried out in a 180 μl reaction mixture. The final concentration of purified ZgHAD protein was 0.3 mg ml^–1^. The kinetics of the reaction was followed by measuring the decrease of absorbance at 560 nm for 10 min on a Spark^®^ multimode microplate reader (Tecan Group Ltd., Switzerland). A standard curve was produced by mixing the assay solution with 1 M HCl to final concentrations of 0–2 mM in a total volume of 200 μl. The Lineweaver-Burke plots were used to calculate the Km and Vmax steady-state kinetic parameters.

### Deletion of ZgHAD Gene in *Zobellia galactanivorans*

A deletion mutant of the *zghad* gene was constructed following the previously described method from Zhu et al., 2017. A 2.2-kb fragment corresponding to the first 36 bp of the *zghad* gene with the region directly upstream was amplified using the primers OEG007 and OEG009 (Supplementary Table 6). The fragment was digested with *Xba*I and *Sal*I and ligated into the pYT313 vector cut with the same enzymes to generate the vector pEG1. Similarly, a 2.3-kb fragment corresponding to the last 35 bp of the *zghad* gene and the region directly downstream was amplified with the primers OEG008 and OEG010 (Supplementary Table 6). The fragment was inserted between *Bam*HI and *Xba*I sites of pEG1 to generate the suicide plasmid pEG3. Suicide plasmid was introduced into the wild type *Z. galactanivorans* Dsij^T^ by conjugation with *E. coli* S17-1 strain. Erythromycin resistance was used to select the cells having integrated the plasmid in their genome. Resulting colonies were grown overnight in *Cytophaga* medium (per liter: 1.0 g yeast extract, 1.0 g tryptone, 24.7 g/NaCl, 0.7 g KCl, 6.3 g MgSO_4_.7H_2_O, 4.6 g MgCl_2_.6H_2_O, 1.2 g CaCl_2_.2H_2_O, 0.2 g NaHCO_3_) without antibiotics at 30°C to allow the loss of the plasmid. The cells were then plated onto *Cytophaga*-agar containing 5% sucrose. The sucrose-resistant colonies were checked for erythromycin sensitivity. The deletion of the *zghad* gene was confirmed by PCR using primer pairs OEG011-OEG012 (Supplementary Table 6) and then verified by Sanger sequencing at the GENOMER platform (FR2424; Roscoff Biological Station).

### Growth Inhibition Analyses of Marine *Flavobacteriaceae* and Phenotyping of the *Zobellia galactanivorans Δhad* Mutant

The ability to grow and resist to increasing haloacid concentrations was tested using an antibiogram-like method. *Z. galactanivorans* wild type (WT) and its deleted *had* gene mutant (Δ*had*) were grown on ZoBell 2216-agar plates with a Whatman paper disk at the center. The paper was impregnated with 25 μl solution of either 500 mM BAA or IAA (shown to be *in vitro* substrates of ZgHAD) or Tris-glycine 100 mM pH 8.0 as negative control. The haloacid effect was determined by monitoring the size of the inhibition zone after 72 h at 20°C. This experiment was realized two times independently.

*Zobellia galactanivorans* WT and both *Tenacibaculum* species (*T. discolor* DSM 18842^T^ and *T. gallaicum* DSM 18841^T^) were grown in 3 ml ZoBell 2216 liquid medium with high concentrations of BAA and IAA (2 mM) buffered by 20 mM HEPES buffer pH 7.0 and during 16 h at 20°C. Each medium was initially inoculated at 1/100 dilution with a two-day preculture. *Z. galactanivorans* WT strain and the mutant Δ*had* strain were also grown in ZoBell liquid medium in the same conditions with different concentration ranges of IAA or BAA dissolved in 200 mM HEPES buffer pH 7.0. The final concentrations were defined from 0 to 1.6 mM and 0 to 8 mM, respectively, for IAA and BAA. Each concentration was tested in biological triplicates. Bacterial growth was measured as the mean of OD600 in technical duplicates. Growth rates were calculated from the slopes of the growth curves between 15 and 20 h, during the exponential phase. They were expressed as a percentage of the standard growth rate in ZoBell medium for each corresponding strain.

### Phenotyping of Recombinant *Escherichia coli* Tolerance to Bromoacetic Acid and Iodoacetic Acid

The *E. coli* BL21 expression strains containing the pFO4 vector alone (pFO4-BL21 strain) or recombined with the *zghad* gene (ZgHAD-BL21 strain) were grown in LB medium containing 100 μg ml^–1^ ampicillin and IPTG overnight at 20°C. These precultures were used to inoculate a fresh LB/Ampicillin medium supplemented with 0.5 mM IPTG at 1/100 dilution. Two concentrations distant of 10 folds were tested in triplicates as follows, 0.5 and 5 mM for BAA and 0.16 and 1.6 mM for IAA. After 15 h of incubation at 20°C, the final OD600 was measured and reported to that of an equivalent culture in standard conditions, without haloacids. Growth was expressed as a percentage of the corresponding culture in standard LB/ampicillin medium without addition of haloacid.

### Statistical Analyses

Growth rate values obtained under the different experiments were analyzed using two-way analysis of variance (two-way ANOVA *p* < 0.05). Mean comparisons between conditions and treatments were made using multiple comparisons by Tukey’s *post hoc* tests at *p* < 0.05 using the PAST software version 4.06b (Hammer et al., 2001).

## Data Availability Statement

The datasets presented in this study can be found in online repositories. The names of the repository/repositories and accession number(s) can be found in the article/Supplementary Material.

## Author Contributions

EG and LD conceived and performed the experiments. EG contributed to the majority of the experiments, data collection, and analyses. AG performed the zg*had* gene cloning and the preliminary expression results. EG wrote the manuscript with the support from LD, FT, and CL. FT contributed in the zg*had* gene deletion experiments, IMG/MER experimental design and general discussion. All authors corrected and approved of the final manuscript.

## Conflict of Interest

The authors declare that the research was conducted in the absence of any commercial or financial relationships that could be construed as a potential conflict of interest.

## Publisher’s Note

All claims expressed in this article are solely those of the authors and do not necessarily represent those of their affiliated organizations, or those of the publisher, the editors and the reviewers. Any product that may be evaluated in this article, or claim that may be made by its manufacturer, is not guaranteed or endorsed by the publisher.

## Footnotes

1
https://img.jgi.doe.gov/cgi-bin/mer/main.cgi?section=FindGenesBlast&page=geneSearchBlast

2
http://tara-oceans.mio.osupytheas.fr/ocean-gene-atlas
