# Supplementary material for: Functional Characterization of a L-2-Haloacid Dehalogenase From Zobellia galactanivorans DsijT Suggests a Role in Haloacetic Acid Catabolism and a Wide Distribution in Marine Environments
Source: Front Microbiol. 2021 Sep 21;12:725997. doi: 10.3389/fmicb.2021.725997 (PMC8490876; doi:10.3389/fmicb.2021.725997)
Supplement: Supplementary file 1 [file Data_Sheet_1.PDF]

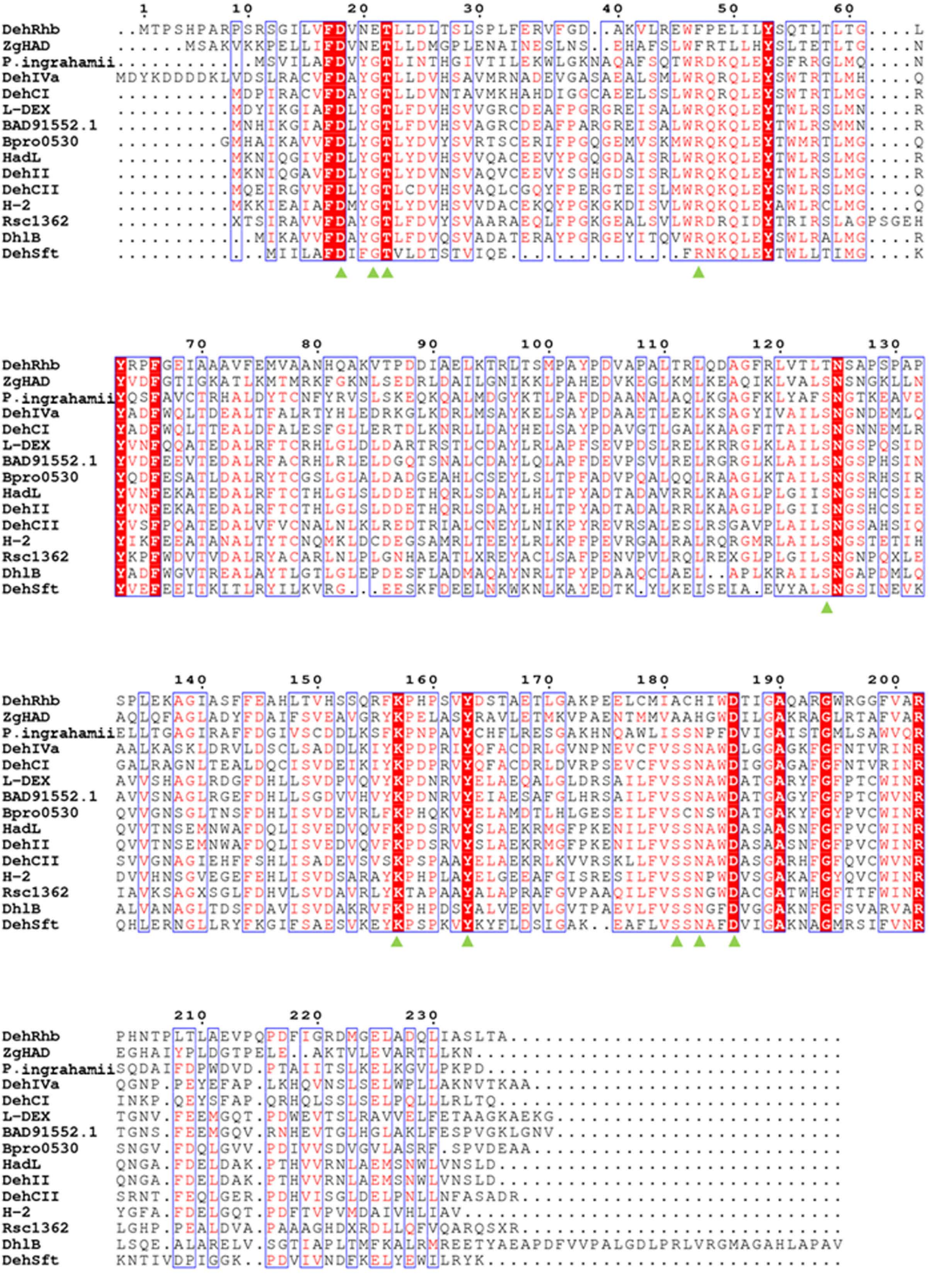

Fig. S1. Amino-acid sequence alignment of ZgHAD and 14 L-2-HAD homologs.
DehPsi from *Psychromonas ingrahamii*, DehRhb from *Rhodobacteraceae*, DehIVa from *Burkholderia cepacia* MBA4, DehCI from *Pseudomonas sp.* CBS3, L-DEX from *Pseudomonas sp.* YL, BAD91552.1 from *Burkholderia sp.* WS, Bpro0530 from *Polaromonas sp.* JS666, HadL from *Pseudomonas putida* AJ1, DehII from *Pseudomonas putida* PP3, DehCII from *Pseudomonas sp.* CBS3, H-2 from *Moraxella sp.* strain B, Rsc1362 from R*alstonia solanacearum* GMI1000, DehlB from *Xanthobacter autotrophicus* and DehSft from *Sulfolobus tokodaii*.
Alignment was realized using the programs MultAlin (Corpet, 1988) and ESPript (Robert and Gouet, 2014). Identical residues are shown in red boxes and residues with similar properties are in blue boxes.
Green arrows show the 10 amino acids essential for catalytic activity and binding substrate.

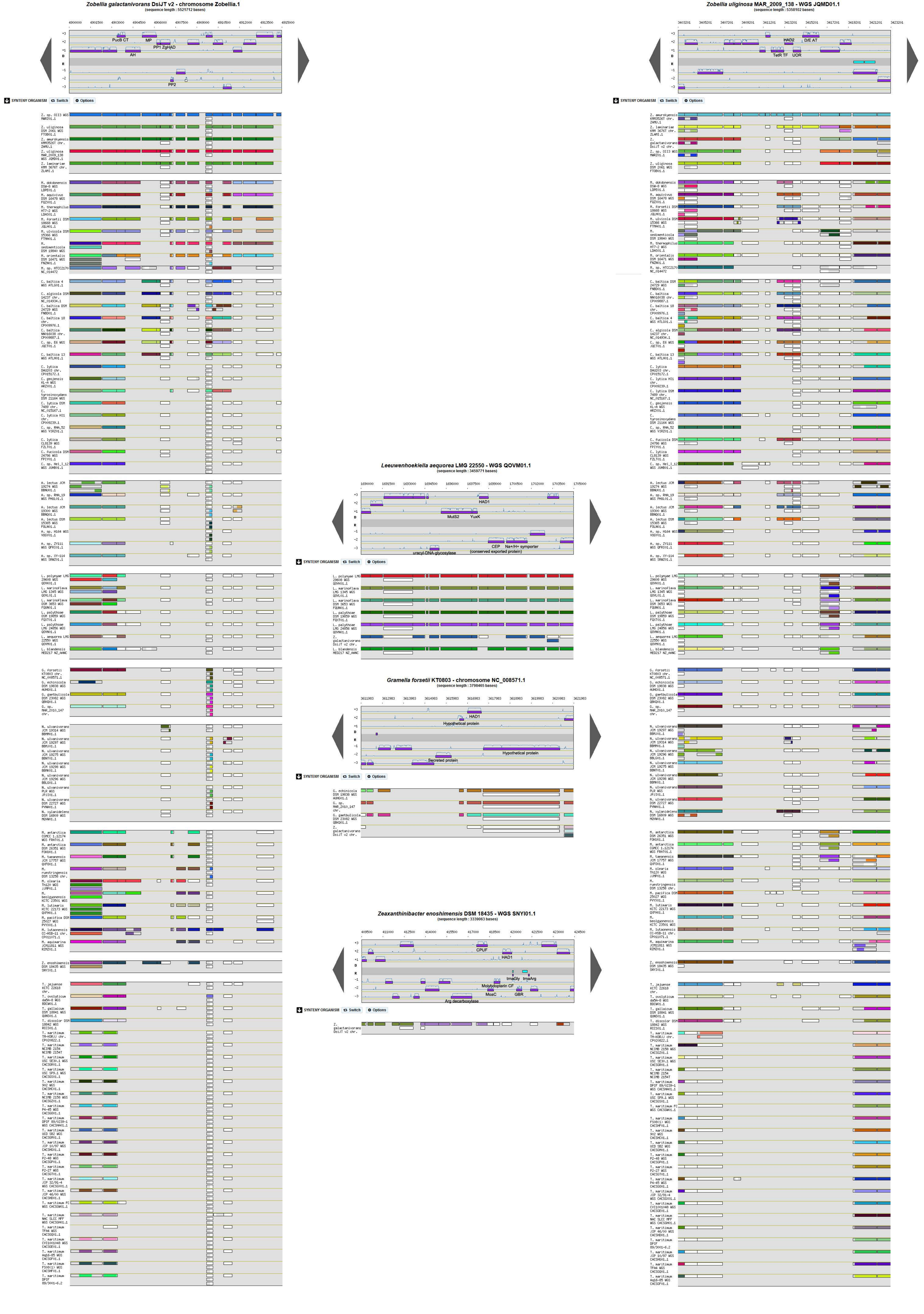

Fig. S2. Detailed analysis of the genetic context for the haloacid dehalogenases HAD1 and HAD2 in *Flavobacteriaceae*.
All the screenshots of synteny overlaps were obtained from the MaGe platform.

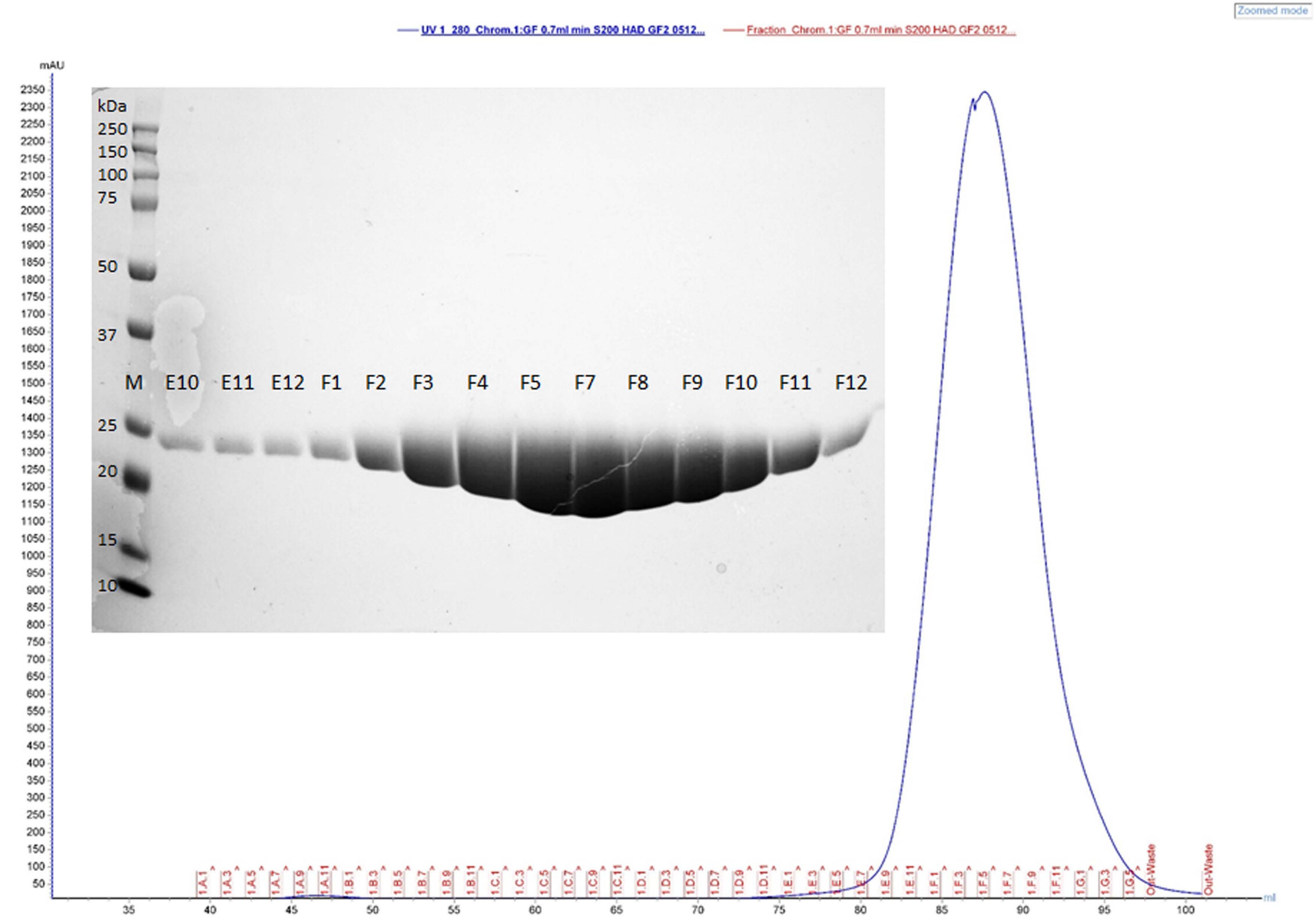

Fig. S3. Chromatogram and SDS-PAGE analyses of ZgHAD purification by size exclusion chromatography.
The polyacrylamide gel was stained with Coomassie Brilliant Blue R-250, revealing the presence of the recombinant protein in eluted fractions of the A280 peak (lanes E10 to F12). M = marker Precision Plus Protein™ Standards (Bio-Rad Laboratories) and the molecular masses are indicated on the left of the lane.

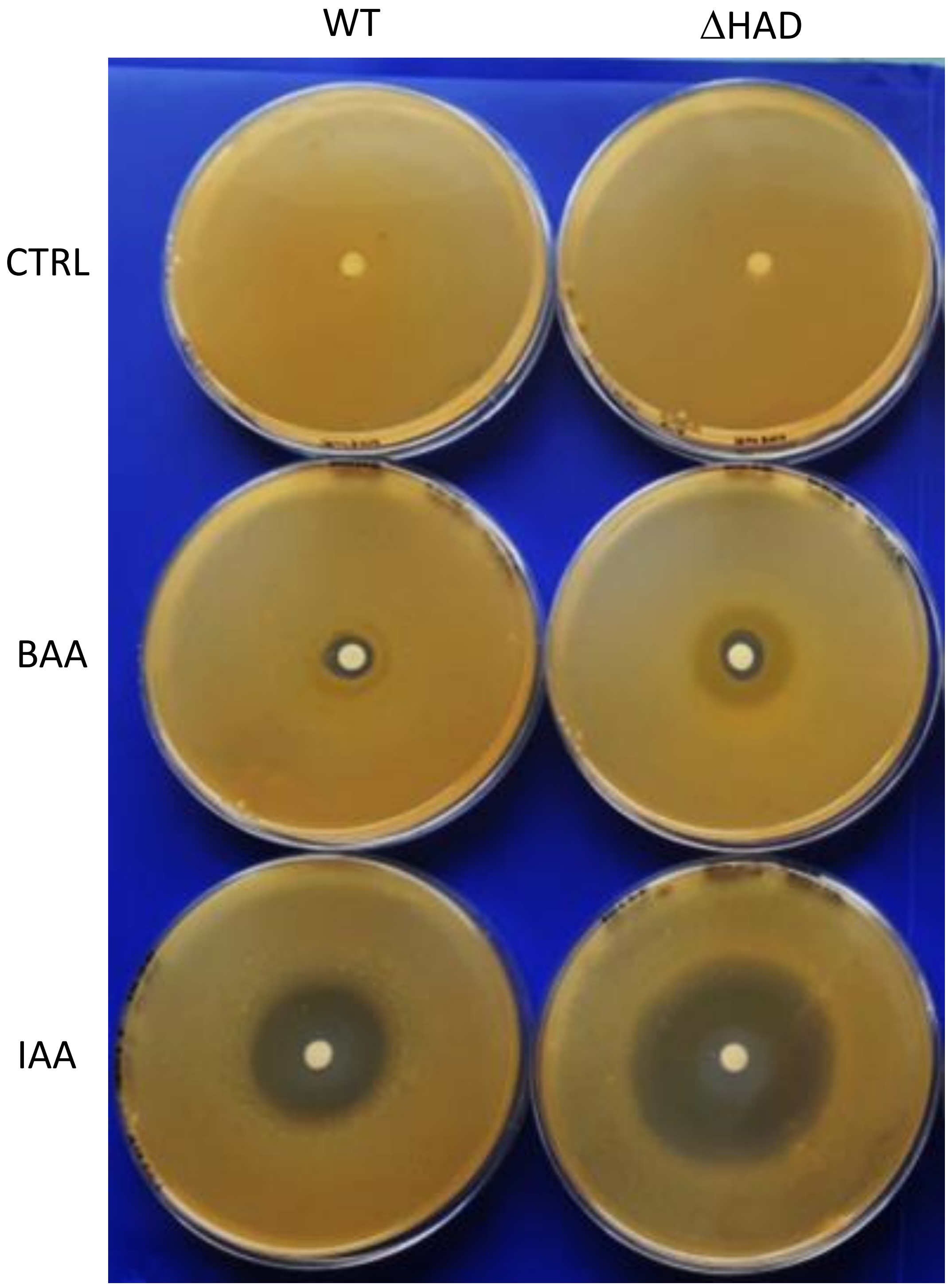

Fig. S4. Second independent replicate of *Z. galactanivorans* WT and *Δhad* growth on solid medium with BAA and IAA solutions deposited on a filter at the center of the plate. A photograph of each plate was taken for after 3 days of incubation at 20 °C. CTRL: negative control.
