# Supplementary material for: Functional Characterization of a L-2-Haloacid Dehalogenase From Zobellia galactanivorans DsijT Suggests a Role in Haloacetic Acid Catabolism and a Wide Distribution in Marine Environments
Source: Front Microbiol. 2021 Sep 21;12:725997. doi: 10.3389/fmicb.2021.725997 (PMC8490876; doi:10.3389/fmicb.2021.725997)
Supplement: Supplementary file 2 [file Data_Sheet_2.PDF]

Supplementary References

Abt, B., Lu, M., Misra, M., Han, C., Nolan, M., Lucas, S., *et al*. (2011) Complete genome sequence of *Cellulophaga algicola* type strain (IC166). Stand Genomic Sci 4: 72-80.

Arun, A.B., Chen, W.M., Lai, W.A., Chao, J.H., Rekha, P.D., Shen, F.T., *et al*. (2009) *Muricauda lutaonensis* sp. nov., a moderate thermophile isolated from a coastal hot spring. Int J Syst Evol Microbiol 59: 2738-2742.

Asahina, A.Y., and Hadfield, M.G. (2014) Complete genome sequence of *Cellulophaga lytica* HI1 using PacBio single-molecule real-time sequencing. Genome Announc 2: 6–7.

Asker, D., Beppu, T., and Ueda, K. (2007) *Zeaxanthinibacter enoshimensis* gen. nov., sp. nov., a novel zeaxanthin-producing marine bacterium of the family *Flavobacteriaceae*, isolated from seawater off Enoshima Island, Japan. Int J Syst Evol Microbiol 57: 837-843.

Barbeyron, T., Carpentier, F., L'haridon, S., Schüler, M., Michel, G., and Amann, R. (2008) Description of *Maribacter forsetii* sp. nov., a marine *Flavobacteriaceae* isolated from North Sea water, and emended description of the genus *Maribacter*. Int J Syst Evol Microbiol 58: 790-797.

Barth, P.T., Bolton, L., and Thomson, J.C. (1992) Cloning and partial sequencing of an operon encoding two *Pseudomonas putida* haloalkanoate dehalogenases of opposite stereospecificity. J Bacteriol 174: 2612-2619.

Bauer, M., Kube, M., Teeling, H., Richter, M., Lombardot, T., Allers, E., *et al*. (1996) Cloning, sequencing and expression in *Escherichia coli* of two *Rhizobium sp.* genes encoding haloalkanoate dehalogenases of opposite stereospecificity. Eur J Biochem 235: 744-749.

Chan, W.Y., Wong, M., Guthrie, J., Savchenko, A.V., Yakunin, A.F., Pai, E.F., *et al*. (2010) Sequence- and activity-based screening of microbial genomes for novel dehalogenases. Microb Biotechnol 3: 107-120.

Chapelais-Baron, M., Goubet, I., Duchaud, E., and Rosenfeld, E. (2017) Draft Genome Sequence of the Iridescent Marine Bacterium *Cellulophaga lytica* CECT 8139. Am Soc Microbiol 5: 1–3.

Chen, J., Li, Y., Zhang, K., and Wang, H. (2018) Whole-Genome Sequence of Phage-Resistant Strain *Escherichia coli* DH5alpha. Genome Announc 6:e00097-18.

Choudhuri, J.V., Meyer, F., Reinhardt, R., Amann, R.I., and Glöckner, F.O. (2006) Whole genome analysis of the marine Bacteroidetes *'Gramella forsetii'* reveals adaptations to degradation of polymeric organic matter. Environ Microbiol 8: 2201-2213.

Corpet, F. (1988) Multiple sequence alignment with hierarchical clustering. Nucleic Acids Res 16: 10881-10890.

De Lorenzo, V., and Timmis, K.N. (1994) Analysis and construction of stable phenotypes in gram-negative bacteria with Tn5- and Tn10-derived minitransposons. Methods Enzymol 235: 386-405.

Groisillier, A., Hervé, C., Jeudy, A., Rebuffet, E., Pluchon, P.F., Chevolot, Y., *et al*. (2010) MARINE-EXPRESS: taking advantage of high throughput cloning and expression strategies for the post-genomic analysis of marine organisms. Microb Cell Fact 9: 45.

Harms, H., Poehlein, A., Thürmer, A., König, G.M., and Schäberle, T.F. (2017) Draft Genome Sequence of *Zobellia sp.* Strain OII3, Isolated from the Coastal Zone of the Baltic Sea. Genome Announc 5: e00737-17.

Hu, J., Wang, F., Han, S.B., Wu, S.L., Wu, M., and Xu, X.W. (2015) Genome sequence of facultatively anaerobic marine bacterium *Maribacter thermophilus* strain HT7-2(T). Mar Genomics 3:265-268.

Huntemann, M., Teshima, H., Lapidus, A., Nolan, M., Lucas, S., Hammon, N., *et al*. (2012) Complete genome sequence of the facultatively anaerobic, appendaged bacterium *Muricauda ruestringensis* type strain (B1(T)). Stand Genomic Sci 6: 185-193.

Hwang, C.Y., Kim, M.H., Bae, G.D., Zhang, G.I., Kim, Y.H., and Cho, B.C. (2009) *Muricauda olearia* sp. nov., isolated from crude-oil-contaminated seawater, and emended description of the genus *Muricauda*. Int J Syst Evol Microbiol 59: 1856-1861.

Jeong, H., Barbe, V., Lee, C.H., Vallenet, D., Yu, D.S., Choi, S.H., *et al*. (2009) Genome sequences of *Escherichia coli* B strains REL606 and BL21(DE3). J Mol Biol 394: 644-652.

Johansen, J.E., Nielsen, P., and Sjøholm, C. (1999) Description of *Cellulophaga baltica* gen. nov., sp. nov. and *Cellulophaga fucicola* gen. nov., sp. nov. and reclassification of [*Cytophaga*] *lytica* to *Cellulophaga lytica* gen. nov., comb. nov. Int J Syst Bacteriol 49: 1231-1240.

Jones, D.H., Barth, P.T., Byrom, D., and Thomas, C.M. (1992) Nucleotide sequence of the structural gene encoding a 2-haloalkanoic acid dehalogenase of *Pseudomonas putida* strain AJ1 and purification of the encoded protein. J Gen Microbiol 138: 675-683.

Kahng, H-Y., Chung, B.S., Lee, D-H., Jung, J.S., Park, J.H., and Jeon, C.O. (2009) *Cellulophaga tyrosinoxydans* sp. nov., a tyrosinase-producing bacterium isolated from seawater. Int J Syst Evol Microbiol 59: 654-657.

Kawasaki, H., Tsuda, K., Matsushita, I., and Tonomura, K. (1992) Lack of homology between two haloacetate dehalogenase genes encoded on a plasmid from *Moraxella sp.* strain B. J Gen Microbiol 138:1317-1323.

Kawasaki, H., Toyama, T., Maeda, T., Nishino, H., and Tonomura, K. (1994) Cloning and sequence analysis of a plasmid-encoded 2-haloacid dehalogenase gene from *Pseudomonas putida* No. 109. Biosci Biotechnol Biochem 58: 160-163.

Kim, J.M., Jin, H.M., and Jeon, CO. (2013) *Muricauda taeanensis* sp. nov., isolated from a marine tidal flat. Int J Syst Evol Microbiol 63: 2672-2677.

Köhler, R., Brokamp, A., Schwarze, R., Reiting, R.H., and Schmidt, F.R. (1998) Characteristics and DNA-sequence of a cryptic haloalkanoic acid dehalogenase from *Agrobacterium tumefaciens* RS5. Curr Microbiol 36: 96-101.

Kopel, M., Helbert, W., Henrissat, B., Doniger, T., and Banin, E. (2014) Draft Genome Sequence of *Nonlabens ulvanivorans*, an Ulvan-Degrading Bacterium. Genome Announc 2: e00793-14.

Kumar, A., Pillay, B., and Olaniran, A.O. L-2-Haloacid dehalogenase from *Ancylobacter aquaticus* UV5: Sequence determination and structure prediction. Int J Biol Macromol 83: 216-225.

Kurata, A., Kurihara, T., Kamachi, H., and Esaki, N. (2005) 2-Haloacrylate reductase, a novel enzyme of the medium chain dehydrogenase/reductase superfamily that catalyzes the reduction of a carbon-carbon double bond of unsaturated organohalogen compounds. J Biol Chem 280: 20286-20291.

Kwak, M-J., Lee, J., Kwon, S-W., and Kim, J.F. (2017) Genome Information of *Maribacter dokdonensis* DSW-8 and Comparative Analysis with Other *Maribacter* Genomes. J Microbiol Biotechnol 27: 591-597.

Lafleur, J.E., Costa, S.K., Bitzer, A.S., and Silby, M.W. (2015) Draft Genome Sequence of *Cellulophaga sp.* E6, a Marine Algal Epibiont That Produces a Quorum-Sensing Inhibitory Compound Active against *Pseudomonas aeruginosa*.

Lee, S.Y., Park, S., Oh, T.K., and Yoon, J.H. (2012) *Muricauda beolgyonensis* sp. nov., isolated from a tidal flat. Int J Syst Evol Microbiol 62: 1134-1139.

Murdiyatmo, U., Asmara, W., Tsang, J.S., Baines, A.J., Bull, A.T., and Hardman, D.J. (1992) Molecular biology of the 2-haloacid halidohydrolase IVa from *Pseudomonas cepacia* MBA4. Biochem J 284: 87-93.

Lee, Y.S., and Choi, Y.L. (2017) Complete genome sequence and analysis of three kinds of β-agarase of *Cellulophaga lytica* DAU203 isolated from marine sediment. Mar Genomics 35: 43–46.

Nakanishi, M., Meirelles, P., Suzuki, R., Takatani, N., Mino, S., Suda, W., *et al.* (2014) Draft Genome Sequences of Marine Flavobacterium *Nonlabens* Strains NR17, NR24, NR27, NR32, NR33, and Ara13. Genome Announc 2: e01165-14.

Nedashkovskaya, O.I., Kim, S.B., Han, S.K., Lysenko, A.M., Rohde, M., Rhee, M.S., *et al.* (2004b) *Maribacter* gen. nov., a new member of the family *Flavobacteriaceae*, isolated from marine habitats, containing the species *Maribacter sedimenticola* sp. nov., *Maribacter aquivirus* sp. nov., *Maribacter orientalis* sp. nov. and *Maribacter ulvicola* sp. nov. Int J Syst Evol Microbiol 54: 1017–1023.

Nedashkovskaya, O.I., Kim, S.B., Han, S.K., Rhee, M.S., Lysenko, A.M., Rohde, M., *et al.* (2004c) *Algibacter lectus* gen. nov., sp. nov., a novel member of the family *Flavobacteriaceae* isolated from green algae. Int J Syst Evol Microbiol 54: 1257–1261.

Nedashkovskaya, O.I., Vancanneyt, M., Dawyndt, P., Engelbeen, K., Vandemeulebroecke, K., Cleenwerck, I., *et al.* (2005) Reclassification of [*Cytophaga*] marinoflava Reichenbach 1989 as *Leeuwenhoekiella marinoflava* gen. nov., comb. nov. and description of *Leeuwenhoekiella aequorea* sp. nov. Int J Syst Evol Microbiol 55: 1033–1038.

Nedashkovskaya, O.I., Vancanneyt, M., Kim, S.B., Zhukova, N.V., Han, J.H., and Mikhailov, V.V. (2009) *Leeuwenhoekiella palythoae* sp. nov., a new member of the family *Flavobacteriaceae*. Int J Syst Evol Microbiol 59: 3074-3077.

Nedashkovskaya, O.I., Kukhlevskiy, A.D., Zhukova, N.V., and Kim, S.B. (2014) *Flavimarina pacifica* gen. nov., sp. nov., a new marine bacterium of the family *Flavobacteriaceae*, and emended descriptions of the genus *Leeuwenhoekiella*, *Leeuwenhoekiella aequorea* and *Leeuwenhoekiella marinoflava*. Antonie Van Leeuwenhoek 106: 421-429.

Oh, H-M., Kang, I., Yang, S-J., Jang, Y., Vergin, K.L., Giovannoni, S.J., and Cho, J-C. (2011) Complete genome sequence of strain HTCC2170, a novel member of the genus *Maribacter* in the family *Flavobacteriaceae*. J Bacteriol 193: 303-304.

Oh, Y.S., Kahng, H.Y., Lee, D.H., and Lee, S.B. (2012) *Tenacibaculum jejuense* sp. nov., isolated from coastal seawater. Int J Syst Evol Microbiol 62: 414-419.

Panschin, I., Huang, S., Meier-Kolthoff, J.P., Tindall, B.J., Rohde, M., Verbarg, S., *et al.* (2016) Comparing polysaccharide decomposition between the type strains *Gramella echinicola* KMM 6050(T) (DSM 19838(T)) and *Gramella portivictoriae* UST040801-

001(T) (DSM 23547(T)), and emended description of *Gramella echinicola* Nedashkovskaya et al. 2005 emend. Shahina et al. 2014 and *Gramella portivictoriae* Lau et al. 2005. Stand Genomic Sci 11: 37.

Panschin, I., Becher, M., Verbarg, S., Spröer, C., Rohde, M., Schüler, M., *et al.* (2017) Description of *Gramella forsetii* sp. nov., a marine *Flavobacteriaceae* isolated from North Sea water, and emended description of *Gramella gaetbulicola* Cho et al. 2011. Int J Syst Evol Microbiol 67: 697-703.

Pati, A., Abt, B., Teshima, H., Nolan, M., Lapidus, A., Lucas, S., *et al.* (2011) Complete genome sequence of *Cellulophaga lytica* type strain (LIM-21). Stand Genomic Sci 4: 221-232.

Piñeiro-Vidal, M., Riaza, A., and Santos, Y. (2008) *Tenacibaculum discolor* sp. nov. and *Tenacibaculum gallaicum* sp. nov., isolated from sole (*Solea senegalensis*) and turbot (*Psetta maxima*) culture systems. Int J Syst Evol Microbiol 58: 21-25.

Pinhassi, J., Bowman, J.P., Nedashkovskaya, O.I., Lekunberri, I., Gomez-Consarnau, L., and Pedrós-Alió, C. (2006) *Leeuwenhoekiella blandensis* sp. nov., a genome-sequenced marine member of the family *Flavobacteriaceae*.

Robert, X. and Gouet, P. (2014) Deciphering key features in protein structures with the new ENDscript server. Nucleic Acids Res 42: W320-W324.

Schneider B, Müller R, Frank R, Lingens F. (1991) Complete nucleotide sequences and comparison of the structural genes of two 2-haloalkanoic acid dehalogenases from *Pseudomonas sp.* strain CBS3. J Bacteriol 173: 1530-1535.

Shan, D., Ying, J., Li, X., Gao, Z., Wei, G., and Shao, Z. (2014) Draft Genome Sequence of the Carrageenan-Degrading Bacterium *Cellulophaga sp.* Strain KL-A, Isolated from Decaying Marine Algae. Genome Announc 2: e00145-14.

Si, O-J., Kim, S-J., Jung, M-Y., Choi, S-B., Kim, J-G., Kim, S-G., *et al*. (2015) *Leeuwenhoekiella polynyae* sp. nov., isolated from a polynya in western Antarctica. Int J Syst Evol Microbiol 65: 1694-1699.

Suzuki, M., Nakagawa, Y., Harayama, S., and Yamamoto, S. (2001) Phylogenetic analysis and taxonomic study of marine *Cytophaga*-like bacteria: proposal for *Tenacibaculum* gen. nov. with *Tenacibaculum maritimum* comb. nov. and *Tenacibaculum ovolyticum* comb. nov., and description of *Tenacibaculum mesophilum* sp. nov. and *Tenacibaculum amylolyticum* sp. nov. Int J Syst Evol Microbiol 51: 1639-1652.

Takatani, N., Nakanishi, M., Meirelles, P., Mino, S., Suda, W., Oshima, K., *et al*. (2014) Draft Genome Sequences of Marine Flavobacterium *Algibacter lectus* Strains SS8 and NR4. Genome Announc 2: e01168-14.

Tully, B.J., Graham, E.D., and Heidelberg, J.F. (2018) The reconstruction of 2,631 draft metagenome-assembled genomes from the global oceans. Sci Data 5: 170203.

Wu, Y.H., Yu, P.S., Zhou, Y.D., Xu, L., Wang, C.S., Wu, M., *et al*. (2013) *Muricauda antarctica* sp. nov., a marine member of the *Flavobacteriaceae* isolated from Antarctic seawater. Int J Syst Evol Microbiol 63: 3451-3456.

Yi, H., and Chun, J. (2012) Unification of the genera *Nonlabens*, *Persicivirga*, *Sandarakinotalea* and *Stenothermobacter* into a single emended genus, *Nonlabens*, and description of *Nonlabens agnitus* sp. nov. Syst Appl Microbiol 35: 150-155.

Yoon, J.H., Lee, M.H., Oh, T.K., Park, Y.H. (2005) *Muricauda flavescens* sp. nov. and *Muricauda aquimarina* sp. nov., isolated from a salt lake near Hwajinpo Beach of the East Sea in Korea, and emended description of the genus *Muricauda*. Int J Syst Evol Microbiol 55: 1015-1019.

Yoon, J.H., Kang, S.J., Jung, Y.T., and Oh, T.K. (2008) *Muricauda lutimaris* sp. nov., isolated from a tidal flat of the Yellow Sea. Int J Syst Evol Microbiol 58: 1603-1607.

Zhang, Z., Gao, X., Qiao, Y., Wang, Y., and Zhang, X.H. (2015) *Muricauda pacifica* sp. nov., isolated from seawater of the South Pacific Gyre. Int J Syst Evol Microbiol 65: 4087-4092.
